# (*E*)-2-(1,3-Diphenyl­allyl­idene)malononitrile

**DOI:** 10.1107/S1600536809048338

**Published:** 2009-11-21

**Authors:** Tai-Ran Kang, Lian-Mei Chen

**Affiliations:** a College of Chemistry and Chemical Engineering, China West Normal University, Nanchong 637002, People’s Republic of China

## Abstract

The title compound, C_18_H_12_N_2_, adopts an *E* conformation with the benzyl­idenemalononitrile and phenyl groups located on opposite sides of the C=C bond. The two phenyl rings are oriented at a dihedral angle of 62.49 (7)°.

## Related literature

For background to the use of malononitrile-containing compounds as building blocks in organic synthesis, see: Erian (1993 ▶); Liu *et al.* (2002 ▶); Sepiol & Milart. (1985 ▶); Zhang *et al.* (2003 ▶). For a related structure, see: Basu Baul *et al.* (2009 ▶).
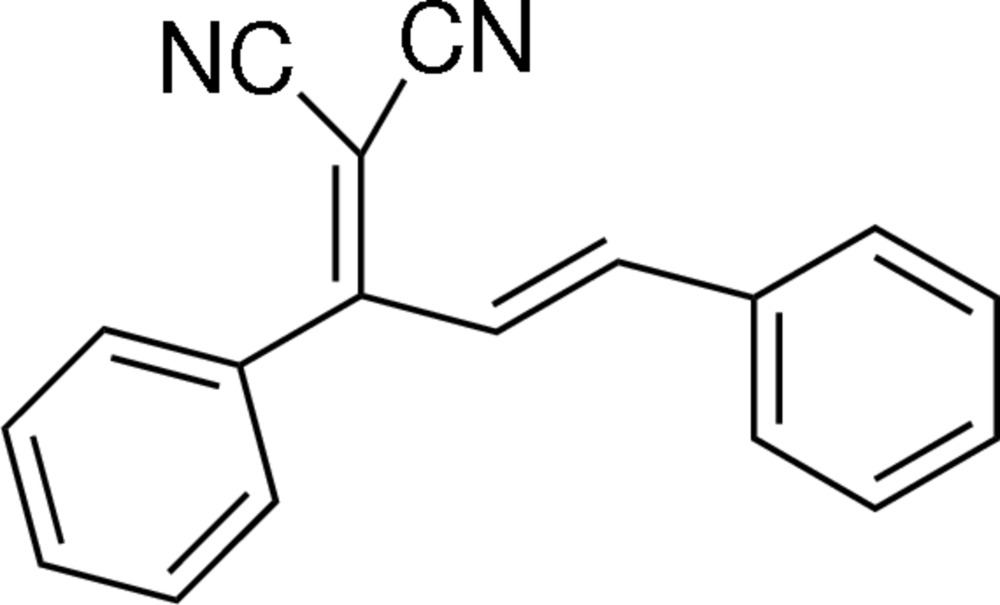


## Experimental

### 

#### Crystal data

C_18_H_12_N_2_
*M* *_r_* = 256.30
Monoclinic, 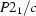

*a* = 12.1658 (6) Å
*b* = 14.8852 (9) Å
*c* = 8.1959 (7) Å
β = 108.457 (6)°
*V* = 1407.85 (16) Å^3^
*Z* = 4
Mo *K*α radiation
μ = 0.07 mm^−1^
*T* = 295 K
0.50 × 0.40 × 0.40 mm

#### Data collection

Oxford Diffraction Gemini S Ultra diffractometer
Absorption correction: none
13329 measured reflections
2880 independent reflections
1735 reflections with *I* > 2σ(*I*)
*R*_int_ = 0.025

#### Refinement

*R*[*F* ^2^ > 2σ(*F* ^2^)] = 0.035
*wR*(*F* ^2^) = 0.082
*S* = 1.01
2880 reflections
181 parameters
3 restraints
H-atom parameters constrained
Δρ_max_ = 0.11 e Å^−3^
Δρ_min_ = −0.14 e Å^−3^

### 

Data collection: *CrysAlis Pro* (Oxford Diffraction, 2009 ▶); cell refinement: *CrysAlis Pro*; data reduction: *CrysAlis Pro*; program(s) used to solve structure: *SHELXS97* (Sheldrick, 2008 ▶); program(s) used to refine structure: *SHELXL97* (Sheldrick, 2008 ▶); molecular graphics: *ORTEP-3* (Farrugia, 1997 ▶); software used to prepare material for publication: *SHELXL97*.

## Supplementary Material

Crystal structure: contains datablocks global, I. DOI: 10.1107/S1600536809048338/xu2673sup1.cif

Structure factors: contains datablocks I. DOI: 10.1107/S1600536809048338/xu2673Isup2.hkl

Additional supplementary materials:  crystallographic information; 3D view; checkCIF report

## Figures and Tables

**Fig. 1.**
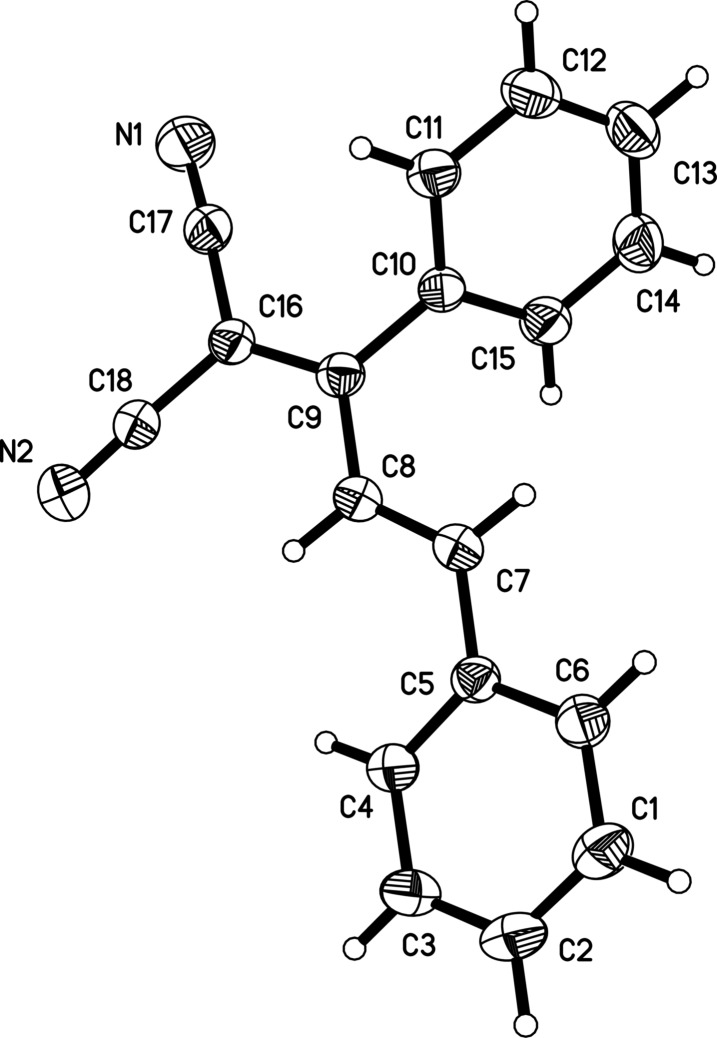
The molecular structure of (I) with 30% probability displacement ellipsoids (arbitrary spheres for H atoms).

## supplementary crystallographic information

### Comment

The chemistry of ylidene malononitrile was studied extensively in organic synthesis (Erian *et al.*, 1993). From the ring closure reactions, the comounds containing newly formed five or six-membered rings, such as indans (Zhang *et al.*, 2003), naphthalenes (Liu, *et al.*, 2002), benzenes (Sepiol & Milart., 1985) were obtained. As a part of our interest in the synthesis of some complex ring systems, we investigated the title compound, (I), which is a diene reagent in the Diels–Alder reaction. We report herein the crystal structure of the title compound.

The molecular structure of (I) is shown in Fig. 1. Bond lengths and angles in (I) are normal. The title compound adopts an E conformation with respect to the C=C bond, the dihedral angle between the C1—C6 phenyl ring and C10—C15 phenyl ring is 62.49 (7)°.

### Experimental

2-(1-Phenylethylidene)malononitrile (0.334 g, 2.14 mmol), benzaldehyde (0.249 g, 2.35 mmol), piperidine (0.018 g, 0.214 mmol) were stirred in 2-propanol (2 ml) at 343 K for 24 h. Then the reaction was cooled to room temperature, and the solution was filtered to obtain a yellow solid. Recrystallization from hot ethanol afforded the pure compound. Single crystals of (I) suitable for X-ray analysis were obtained by slow evaporation ethanol solvent.

### Refinement

The carbon-bound hydrogen atoms were placed in calculated positions, with C—H = 0.93 Å, and refined using a riding model, with *U*_iso_(H) =1.2*U*_eq_(C).

### Crystal data

**Table:**

C_18_H_12_N_2_	*F*(000) = 536
*M**_r_* = 256.30	*D*_x_ = 1.209 Mg m^−^^3^
Monoclinic, *P*2_1_/*c*	Mo *K*α radiation, λ = 0.71073 Å
Hall symbol: -P 2ybc	Cell parameters from 5219 reflections
*a* = 12.1658 (6) Å	θ = 3.0–29.2°
*b* = 14.8852 (9) Å	µ = 0.07 mm^−^^1^
*c* = 8.1959 (7) Å	*T* = 295 K
β = 108.457 (6)°	Block, yellow
*V* = 1407.85 (16) Å^3^	0.50 × 0.40 × 0.40 mm
*Z* = 4	

### Data collection

**Table:**

Oxford Diffraction Gemini S Ultra diffractometer	1735 reflections with *I* > 2σ(*I*)
Radiation source: Enhance (Mo) X-ray Source	*R*_int_ = 0.025
graphite	θ_max_ = 26.4°, θ_min_ = 3.0°
Detector resolution: 15.9149 pixels mm^-1^	*h* = −15→15
ω scans	*k* = −12→18
13329 measured reflections	*l* = −10→10
2880 independent reflections	

### Refinement

**Table:**

Refinement on *F*^2^	Primary atom site location: structure-invariant direct methods
Least-squares matrix: full	Secondary atom site location: difference Fourier map
*R*[*F*^2^ > 2σ(*F*^2^)] = 0.035	Hydrogen site location: inferred from neighbouring sites
*wR*(*F*^2^) = 0.082	H-atom parameters constrained
*S* = 1.01	*w* = 1/[σ^2^(*F*_o_^2^) + (0.0388*P*)^2^] where *P* = (*F*_o_^2^ + 2*F*_c_^2^)/3
2880 reflections	(Δ/σ)_max_ = 0.001
181 parameters	Δρ_max_ = 0.11 e Å^−^^3^
3 restraints	Δρ_min_ = −0.13 e Å^−^^3^

### Special details

**Table:**

Geometry. All e.s.d.'s (except the e.s.d. in the dihedral angle between two l.s. planes) are estimated using the full covariance matrix. The cell e.s.d.'s are taken into account individually in the estimation of e.s.d.'s in distances, angles and torsion angles; correlations between e.s.d.'s in cell parameters are only used when they are defined by crystal symmetry. An approximate (isotropic) treatment of cell e.s.d.'s is used for estimating e.s.d.'s involving l.s. planes.
Refinement. Refinement of *F*^2^ against ALL reflections. The weighted *R*-factor *wR* and goodness of fit *S* are based on *F*^2^, conventional *R*-factors *R* are based on *F*, with *F* set to zero for negative *F*^2^. The threshold expression of *F*^2^ > σ(*F*^2^) is used only for calculating *R*-factors(gt) *etc*. and is not relevant to the choice of reflections for refinement. *R*-factors based on *F*^2^ are statistically about twice as large as those based on *F*, and *R*- factors based on ALL data will be even larger.

### Fractional atomic coordinates and isotropic or equivalent isotropic displacement parameters (Å^2^)

**Table:**

	*x*	*y*	*z*	*U*_iso_*/*U*_eq_	
C8	1.02337 (9)	0.42702 (8)	0.80790 (16)	0.0520 (3)	
H8	0.9756	0.4731	0.7487	0.062*	
C9	1.14221 (9)	0.42886 (8)	0.80802 (15)	0.0468 (3)	
C16	1.17980 (10)	0.50107 (7)	0.73655 (16)	0.0492 (3)	
C10	1.22053 (10)	0.35243 (7)	0.87880 (15)	0.0466 (3)	
C7	0.97483 (10)	0.36634 (8)	0.88354 (15)	0.0492 (3)	
H7	1.0225	0.3211	0.9464	0.059*	
C4	0.76990 (10)	0.41976 (8)	0.76497 (17)	0.0578 (3)	
H4	0.7914	0.4586	0.6915	0.069*	
C6	0.81719 (11)	0.30629 (8)	0.98007 (17)	0.0564 (3)	
H6	0.8706	0.2677	1.0531	0.068*	
C5	0.85340 (9)	0.36488 (7)	0.87593 (15)	0.0461 (3)	
C1	0.70315 (12)	0.30433 (9)	0.97724 (19)	0.0679 (4)	
H1	0.6804	0.2649	1.0484	0.081*	
C2	0.62347 (11)	0.36058 (9)	0.86948 (19)	0.0679 (4)	
H2	0.5470	0.3601	0.8692	0.082*	
C11	1.32866 (10)	0.36506 (8)	0.99982 (17)	0.0572 (3)	
H11	1.3530	0.4227	1.0384	0.069*	
C3	0.65660 (11)	0.41749 (9)	0.76217 (18)	0.0651 (4)	
H3	0.6021	0.4547	0.6873	0.078*	
C17	1.29300 (11)	0.50807 (8)	0.71970 (17)	0.0578 (3)	
C14	1.25937 (13)	0.19431 (9)	0.8861 (2)	0.0751 (4)	
H14	1.2363	0.1364	0.8474	0.090*	
C15	1.18644 (11)	0.26573 (8)	0.82240 (18)	0.0604 (4)	
H15	1.1142	0.2559	0.7414	0.073*	
C13	1.36577 (13)	0.20804 (10)	1.0064 (2)	0.0778 (5)	
H13	1.4144	0.1595	1.0492	0.093*	
C12	1.40048 (12)	0.29275 (10)	1.06346 (19)	0.0707 (4)	
H12	1.4726	0.3018	1.1453	0.085*	
C18	1.10284 (11)	0.57361 (9)	0.66242 (17)	0.0544 (3)	
N2	1.03886 (10)	0.62944 (8)	0.60031 (16)	0.0742 (4)	
N1	1.38156 (11)	0.51654 (8)	0.70138 (18)	0.0851 (4)	

### Atomic displacement parameters (Å^2^)

**Table:**

	*U*^11^	*U*^22^	*U*^33^	*U*^12^	*U*^13^	*U*^23^
C8	0.0442 (7)	0.0488 (7)	0.0631 (8)	0.0040 (5)	0.0171 (6)	0.0015 (6)
C9	0.0427 (7)	0.0482 (7)	0.0495 (8)	−0.0006 (5)	0.0144 (5)	−0.0057 (6)
C16	0.0458 (6)	0.0452 (7)	0.0570 (8)	−0.0010 (5)	0.0169 (6)	−0.0030 (6)
C10	0.0433 (7)	0.0475 (7)	0.0530 (8)	0.0017 (5)	0.0208 (6)	0.0013 (6)
C7	0.0462 (7)	0.0503 (7)	0.0505 (8)	0.0049 (5)	0.0146 (6)	0.0000 (6)
C4	0.0474 (7)	0.0592 (8)	0.0668 (9)	0.0011 (6)	0.0178 (6)	0.0085 (7)
C6	0.0566 (8)	0.0565 (7)	0.0588 (9)	0.0020 (6)	0.0222 (7)	0.0023 (7)
C5	0.0440 (7)	0.0458 (7)	0.0508 (8)	0.0001 (5)	0.0182 (6)	−0.0039 (6)
C1	0.0608 (9)	0.0757 (9)	0.0755 (11)	−0.0076 (7)	0.0335 (8)	0.0056 (8)
C2	0.0465 (8)	0.0847 (10)	0.0779 (10)	−0.0075 (7)	0.0272 (7)	−0.0097 (9)
C11	0.0486 (7)	0.0614 (8)	0.0613 (9)	−0.0012 (6)	0.0168 (6)	0.0034 (7)
C3	0.0436 (8)	0.0732 (9)	0.0757 (10)	0.0054 (6)	0.0149 (7)	0.0012 (8)
C17	0.0549 (7)	0.0536 (8)	0.0690 (9)	−0.0005 (6)	0.0253 (7)	0.0041 (7)
C14	0.0774 (11)	0.0503 (8)	0.1059 (13)	0.0079 (7)	0.0408 (10)	0.0020 (8)
C15	0.0530 (8)	0.0548 (8)	0.0753 (10)	0.0018 (6)	0.0229 (7)	−0.0052 (7)
C13	0.0682 (10)	0.0724 (11)	0.0999 (13)	0.0228 (8)	0.0365 (10)	0.0281 (9)
C12	0.0509 (8)	0.0846 (11)	0.0754 (11)	0.0103 (7)	0.0181 (7)	0.0199 (9)
C18	0.0528 (7)	0.0489 (7)	0.0626 (9)	−0.0029 (5)	0.0199 (6)	0.0020 (6)
N2	0.0721 (8)	0.0606 (7)	0.0865 (10)	0.0055 (6)	0.0202 (7)	0.0097 (7)
N1	0.0661 (8)	0.0863 (9)	0.1148 (11)	0.0025 (6)	0.0457 (8)	0.0183 (8)

### Geometric parameters (Å, °)

**Table:**

C8—C7	1.3344 (15)	C1—C2	1.3706 (19)
C8—C9	1.4457 (14)	C1—H1	0.9300
C8—H8	0.9300	C2—C3	1.3703 (18)
C9—C16	1.3701 (15)	C2—H2	0.9300
C9—C10	1.4795 (15)	C11—C12	1.3804 (17)
C16—C17	1.4308 (16)	C11—H11	0.9300
C16—C18	1.4323 (18)	C3—H3	0.9300
C10—C11	1.3869 (16)	C17—N1	1.1409 (14)
C10—C15	1.3892 (16)	C14—C13	1.372 (2)
C7—C5	1.4590 (15)	C14—C15	1.3773 (18)
C7—H7	0.9300	C14—H14	0.9300
C4—C3	1.3718 (16)	C15—H15	0.9300
C4—C5	1.3932 (16)	C13—C12	1.364 (2)
C4—H4	0.9300	C13—H13	0.9300
C6—C1	1.3805 (16)	C12—H12	0.9300
C6—C5	1.3861 (15)	C18—N2	1.1423 (15)
C6—H6	0.9300		
			
C7—C8—C9	127.01 (11)	C2—C1—H1	120.0
C7—C8—H8	116.5	C6—C1—H1	120.0
C9—C8—H8	116.5	C3—C2—C1	119.97 (12)
C16—C9—C8	118.92 (10)	C3—C2—H2	120.0
C16—C9—C10	120.68 (10)	C1—C2—H2	120.0
C8—C9—C10	120.36 (10)	C12—C11—C10	120.54 (12)
C9—C16—C17	124.07 (10)	C12—C11—H11	119.7
C9—C16—C18	120.73 (10)	C10—C11—H11	119.7
C17—C16—C18	115.09 (10)	C2—C3—C4	120.18 (12)
C11—C10—C15	118.66 (11)	C2—C3—H3	119.9
C11—C10—C9	121.57 (11)	C4—C3—H3	119.9
C15—C10—C9	119.77 (11)	N1—C17—C16	177.09 (14)
C8—C7—C5	125.53 (11)	C13—C14—C15	120.38 (13)
C8—C7—H7	117.2	C13—C14—H14	119.8
C5—C7—H7	117.2	C15—C14—H14	119.8
C3—C4—C5	121.17 (12)	C14—C15—C10	120.15 (13)
C3—C4—H4	119.4	C14—C15—H15	119.9
C5—C4—H4	119.4	C10—C15—H15	119.9
C1—C6—C5	121.10 (12)	C12—C13—C14	120.22 (13)
C1—C6—H6	119.5	C12—C13—H13	119.9
C5—C6—H6	119.5	C14—C13—H13	119.9
C6—C5—C4	117.56 (10)	C13—C12—C11	120.06 (14)
C6—C5—C7	119.86 (11)	C13—C12—H12	120.0
C4—C5—C7	122.57 (11)	C11—C12—H12	120.0
C2—C1—C6	119.99 (12)	N2—C18—C16	177.74 (14)
			
C7—C8—C9—C16	−174.10 (11)	C8—C7—C5—C6	−170.60 (11)
C7—C8—C9—C10	8.02 (18)	C8—C7—C5—C4	10.37 (18)
C8—C9—C16—C17	−176.08 (12)	C5—C6—C1—C2	0.38 (19)
C10—C9—C16—C17	1.80 (17)	C6—C1—C2—C3	1.3 (2)
C8—C9—C16—C18	−0.14 (17)	C15—C10—C11—C12	−0.38 (18)
C10—C9—C16—C18	177.74 (12)	C9—C10—C11—C12	−179.70 (11)
C16—C9—C10—C11	53.38 (16)	C1—C2—C3—C4	−1.44 (19)
C8—C9—C10—C11	−128.77 (12)	C5—C4—C3—C2	−0.05 (19)
C16—C9—C10—C15	−125.93 (12)	C13—C14—C15—C10	0.4 (2)
C8—C9—C10—C15	51.92 (15)	C11—C10—C15—C14	−0.09 (18)
C9—C8—C7—C5	−177.82 (12)	C9—C10—C15—C14	179.25 (11)
C1—C6—C5—C4	−1.80 (17)	C15—C14—C13—C12	−0.3 (2)
C1—C6—C5—C7	179.11 (12)	C14—C13—C12—C11	−0.2 (2)
C3—C4—C5—C6	1.64 (17)	C10—C11—C12—C13	0.5 (2)
C3—C4—C5—C7	−179.30 (12)		
